# Completeness of Reporting of Patient-Relevant Clinical Trial Outcomes: Comparison of Unpublished Clinical Study Reports with Publicly Available Data

**DOI:** 10.1371/journal.pmed.1001526

**Published:** 2013-10-08

**Authors:** Beate Wieseler, Natalia Wolfram, Natalie McGauran, Michaela F. Kerekes, Volker Vervölgyi, Petra Kohlepp, Marloes Kamphuis, Ulrich Grouven

**Affiliations:** 1 Institute for Quality and Efficiency in Health Care, Cologne, Germany; 2 Hanover Medical School, Hanover, Germany; National Health & Medical Research Council, Australia

## Abstract

**Background:**

Access to unpublished clinical study reports (CSRs) is currently being discussed as a means to allow unbiased evaluation of clinical research. The Institute for Quality and Efficiency in Health Care (IQWiG) routinely requests CSRs from manufacturers for its drug assessments.

Our objective was to determine the information gain from CSRs compared to publicly available sources (journal publications and registry reports) for patient-relevant outcomes included in IQWiG health technology assessments (HTAs) of drugs.

**Methods and Findings:**

We used a sample of 101 trials with full CSRs received for 16 HTAs of drugs completed by IQWiG between 15 January 2006 and 14 February 2011, and analyzed the CSRs and the publicly available sources of these trials. For each document type we assessed the completeness of information on all patient-relevant outcomes included in the HTAs (benefit outcomes, e.g., mortality, symptoms, and health-related quality of life; harm outcomes, e.g., adverse events). We dichotomized the outcomes as “completely reported” or “incompletely reported.” For each document type, we calculated the proportion of outcomes with complete information per outcome category and overall.

We analyzed 101 trials with CSRs; 86 had at least one publicly available source, 65 at least one journal publication, and 50 a registry report. The trials included 1,080 patient-relevant outcomes. The CSRs provided complete information on a considerably higher proportion of outcomes (86%) than the combined publicly available sources (39%). With the exception of health-related quality of life (57%), CSRs provided complete information on 78% to 100% of the various benefit outcomes (combined publicly available sources: 20% to 53%). CSRs also provided considerably more information on harms. The differences in completeness of information for patient-relevant outcomes between CSRs and journal publications or registry reports (or a combination of both) were statistically significant for all types of outcomes.

The main limitation of our study is that our sample is not representative because only CSRs provided voluntarily by pharmaceutical companies upon request could be assessed. In addition, the sample covered only a limited number of therapeutic areas and was restricted to randomized controlled trials investigating drugs.

**Conclusions:**

In contrast to CSRs, publicly available sources provide insufficient information on patient-relevant outcomes of clinical trials. CSRs should therefore be made publicly available.

*Please see later in the article for the Editors' Summary*

## Abstract 2

Beate Wieseler and colleagues compare the completeness of reporting of patient-relevant clinical trial outcomes between clinical study reports and publicly available data.

*Please see later in the article for the Editors' Summary*

## Editors' Summary

**Background:**

People assume that, when they are ill, health care professionals will ensure that they get the best available treatment. In the past, clinicians used their own experience to make decisions about which treatments to offer their patients, but nowadays, they rely on evidence-based medicine—the systematic review and appraisal of clinical trials, studies that investigate the benefits and harms of drugs and other medical interventions in patients. Evidence-based medicine can guide clinicians, however, only if all the results of clinical research are available for evaluation. Unfortunately, the results of trials in which a new drug performs better than existing drugs are more likely to be published than those in which the new drug performs badly or has unwanted side effects (publication bias). Moreover, trial outcomes that support the use of a new treatment are more likely to be published than those that do not support its use (outcome reporting bias). Both types of bias pose a substantial threat to informed medical decision-making.

**Why Was This Study Done?:**

Recent initiatives, such as making registration of clinical trials in a trial registry (for example, ClinicalTrials.gov) a precondition for publication in medical journals, aim to prevent these biases but are imperfect. Another way to facilitate the unbiased evaluation of clinical research might be to increase access to clinical study reports (CSRs)—detailed but generally unpublished accounts of clinical trials. Notably, information from CSRs was recently used to challenge conclusions based on published evidence about the efficacy and safety of the antiviral drug oseltamivir and the antidepressant reboxetine. In this study, the researchers compare the information available in CSRs and in publicly available sources (journal publications and registry reports) for the patient-relevant outcomes included in 16 health technology assessments (HTAs; analyses of the medical implications of the use of specific medical technologies) for drugs; the HTAs were prepared by the Institute for Quality and Efficiency in Health Care (IQWiG), Germany's main HTA agency.

**What Did the Researchers Do and Find?:**

The researchers searched for published journal articles and registry reports for each of 101 trials for which the IQWiG had requested and received full CSRs from drug manufacturers during HTA preparation. They then assessed the completeness of information on the patient-relevant benefit and harm outcomes (for example symptom relief and adverse effects, respectively) included in each document type. Eighty-six of the included trials had at least one publicly available data source; the results of 15% of the trials were not available in either journals or registry reports. Overall, the CSRs provided complete information on 86% of the patient-related outcomes, whereas the combined publicly available sources provided complete information on only 39% of the outcomes. For individual outcomes, the CSRs provided complete information on 78%–100% of the benefit outcomes, with the exception of health-related quality of life (57%); combined publicly available sources provided complete information on 20%–53% of these outcomes. The CSRs also provided more information on patient-relevant harm outcomes than the publicly available sources.

**What Do These Findings Mean?:**

These findings show that, for the clinical trials considered here, publicly available sources provide much less information on patient-relevant outcomes than CSRs. The generalizability of these findings may be limited, however, because the trials included in this study are not representative of all trials. Specifically, only CSRs that were voluntarily provided by drug companies were assessed, a limited number of therapeutic areas were covered by the trials, and the trials investigated only drugs. Nevertheless, these findings suggest that access to CSRs is important for the unbiased evaluation of clinical trials and for informed decision-making in health care. Notably, in June 2013, the European Medicines Agency released a draft policy calling for the proactive publication of complete clinical trial data (possibly including CSRs). In addition, the European Union and the European Commission are considering legal measures to improve the transparency of clinical trial data. Both these initiatives will probably only apply to drugs that are approved after January 2014, however, and not to drugs already in use. The researchers therefore call for CSRs to be made publicly available for both past and future trials, a recommendation also supported by the AllTrials initiative, which is campaigning for all clinical trials to be registered and fully reported.

**Additional Information:**

Please access these websites via the online version of this summary at http://dx.doi.org/10.1371/journal.pmed.1001526.

- Wikipedia has pages on evidence-based medicine, publication bias, and health technology assessment (note: Wikipedia is a free online encyclopedia that anyone can edit; available in several languages)
- The ClinicalTrials.gov website is a searchable register of federally and privately supported clinical trials in the US; it provides information about all aspects of clinical trials
- The European Medicines Agency (EMA) provides information about all aspects of the scientific evaluation and approval of new medicines in the European Union, and guidance on the preparation of clinical study reports; its draft policy on the release of data from clinical trials is available
- Information about IQWiG is available (in English and German); Informed Health Online is a website provided by IQWiG that provides objective, independent, and evidence-based information for patients (also in English and German)

## Introduction

Publication bias and outcome reporting bias pose a substantial threat to the validity of clinical research findings and thus to informed decision-making in health care [1,2]. In recent years major initiatives to prevent or at least identify these biases have been implemented, such as registration of clinical trials as a precondition for publication in medical journals in 2005 [3], or mandatory trial registration and reporting of methods and results in ClinicalTrials.gov following the Food and Drug Administration Amendments Act of 2007 [4]. However, the application of these measures has been insufficient [5–8], and they also contain several loopholes [9]. For instance, the measures do not apply to clinical trials completed before 2005 and 2007, respectively, and provide only summarized information, preventing full evaluation.

Various types of formats exist for reporting clinical trials of drugs: journal publications and reports from trial registries and results databases—hereafter referred to as “registry reports”—make summaries of trials publicly available (e.g., to clinicians and authors of systematic reviews). These publicly available formats currently represent the main information source for clinical and health policy decision-making. Reporting standards for these two formats include the Consolidated Standards of Reporting Trials (CONSORT [10]) for journal publications and the Food and Drug Administration Amendments Act for registry reports on trials of US Food and Drug Administration–regulated drugs and medical devices [4]. In contrast to the first two formats, clinical study reports (CSRs) are detailed accounts of trials generally prepared following the International Conference on Harmonisation's *Guideline for Industry: Structure and Content of Clinical Study Reports* (ICH E3 [11]). The value of additional information from CSRs in drug assessment has been shown in the cases of the antiviral oseltamivir (Tamiflu) and the antidepressant reboxetine, in which conclusions on these drugs based on published evidence alone were challenged and in part even reversed by unpublished information from CSRs [12,13].

So far, CSRs are used to inform regulatory decision-making, but are in general not publicly available. The few cases in which CSRs have been used for drug evaluation outside regulatory agencies required major efforts by researchers to gain access to the documents [14–16]. However, the European Medicines Agency (EMA) has launched an initiative to improve transparency in clinical research by providing unpublished clinical trial data [17,18]. This initiative also involves a discussion of the data formats to be made publicly available [19], and CSRs are being considered, in addition to individual patient data [20]. Furthermore, legal measures to improve transparency have been proposed by the European Commission and the European Parliament [21,22], also addressing the extent of trial data to be published. Thus, the role of CSRs for the evaluation of clinical trials is currently of particular importance, and we would like to further inform the current debate with our experiences.

### Health Technology Assessments of Drugs at the Institute for Quality and Efficiency in Health Care

The Institute for Quality and Efficiency in Health Care (Institut für Qualität und Wirtschaftlichkeit im Gesundheitswesen; IQWiG), established in 2004, is Germany's main health technology assessment (HTA) agency. Its primary responsibility is the production of HTA reports on drugs and non-drug interventions based on the analysis of patient-relevant outcomes, i.e., outcomes describing morbidity, mortality, and health-related quality of life (HRQoL). These reports inform health policy decision-making in the German statutory health care system. IQWiG attempts to obtain the most complete information possible for its HTAs. For this purpose, during the preparation of a drug report, besides systematically searching bibliographic databases and trial (results) registries, we routinely ask the manufacturer to provide an overview of sponsored published and unpublished clinical trials of the drug under assessment. From this list we select the trials deemed relevant to the assessment and ask the manufacturer to submit the full CSRs. However, except for early assessments of new drugs (which are not the subject of this article), the manufacturer is not obliged to provide CSRs.

### Previous Study of Clinical Study Reports versus Publicly Available Sources

In a previous study investigating the availability of information on methods and selected outcomes of clinical trials in different types of reporting formats, we used the pool of randomized controlled trials and corresponding documents (CSRs, journal publications, registry reports) included in HTAs of drugs prepared by IQWiG (see below). This previous study showed that journal publications and registry reports had different strengths and weaknesses and that, overall, the CSRs provided considerably more complete information on items relating to methods and selected outcomes than publicly available sources [23].

### Rationale for Current Study

The previous study investigated only a limited range of outcomes, i.e., primary outcomes (irrespective of whether they were patient-relevant or not) and some adverse event (AE) outcomes. However, as stated, our HTAs are generally based on a wide range of patient-relevant outcomes (irrespective of whether they are primary outcomes or not). We hypothesized that the information gain from CSRs versus publicly available sources could be even greater for patient-relevant outcomes (which are often non-primary) than for the subset of outcomes investigated in the previous study. In the current study we therefore investigated the information gain for all patient-relevant outcomes included in our HTAs. We also aimed to characterize the information gain from CSRs for various types of patient-relevant outcomes.

## Methods

The methods for the current study were largely based on those described previously [23]. In the previous study, we included all HTAs of drugs finalized by IQWiG between 15 January 2006 and 14 February 2011, which—besides a systematic search for journal publications—contained a systematic search for registry reports as part of the information retrieval process. The systematic search generally covered MEDLINE, Embase, and the databases of the Cochrane Library, as well as ClinicalTrials.gov, the International Clinical Trials Registry Platform of the World Health Organization, the Clinical Trials Portal of the International Federation of Pharmaceutical Manufacturers and Associations, the Clinical Study Results Database of the Pharmaceutical Research and Manufacturers of America, and the trial registries and results databases of the manufacturers of the drugs under investigation. In addition, for all HTAs, CSRs were requested from the manufacturers of the drugs under assessment.

In the previous study we included all 286 trials and corresponding documents (101 CSRs, 192 journal publications, and 78 registry reports) considered in the 16 HTAs. For the current study, we used the same pool of HTAs, but included only the 101 trials from the original pool of 286 trials for which the manufacturer had provided a full CSR. “Full” referred to the availability of a core text (including a full description of methods and results) and all tables and figures, as well as appendices (e.g., protocol or statistical analysis plan) if they were referenced in the core text with only insufficient information provided in the text. None of these CSRs were publicly available at the time of preparation of the HTAs.

As stated, the previous study investigated the reporting of only a limited set of trial outcomes in the various reporting formats. In contrast, our current study aimed to characterize reporting in CSRs versus publicly available documents for all patient-relevant outcomes considered in our HTAs. These outcomes had been prespecified in the HTA protocols during the preparation of the 16 HTAs, and had been identified in the three reporting formats by systematically screening all of the available CSRs, registry reports, and journal publications.

We entered all data for the previous and current study into a Microsoft Access database. The database contained information on the characteristics of the HTA, the type of document available, as well as basic trial and document characteristics (see Table 1). For the current study we also entered data on the reporting quality of all patient-relevant outcomes as described below. In addition, we classified these outcomes as mortality, clinical events, symptoms, or HRQoL (benefit outcomes), as well as AEs, serious AEs (SAEs), AEs of special interest, or withdrawals due to AEs (harm outcomes); please see Table 2 and Box 1 for coding definitions.

**Table 1:** Characteristics of included trials, documents, and outcomes.

Category	Subcategory	Characteristic	Number of Studies or Outcomes (Percent)
**Trial characteristics**	**Trials included**		101 (100)
	**Therapeutic area**	Depression	40 (40)
		Type II diabetes	30 (30)
		Type I diabetes	14 (14)
		Asthma	9 (9)
		Stroke/transient ischemic attack	5 (5)
		Alzheimer disease	3 (3)
	**Phase** a	Premarketing	56 (55)
		Post-marketing	45 (45)
	**Objective**	Efficacy trial^b^	90 (89)
		Safety trial^c^	3 (3)
		Explorative trial	8 (8)
	**Blinding**	Double-blinded	70 (69)
		Open-label	31 (31)
	**Controls**	Placebo only	27 (27)
		Active and placebo	13 (13)
		Active only	61 (60)
	**Funding**	Industry funding	101 (100)
		Non-industry funding	0 (0)
	**Document type available**	Full CSR	101 (100)
		Journal publication	65 (64)
		Registry report	50 (50)
		Report from clinicalstudyresults.org^d^	17 (34)
		Report from company registries	33 (66)
		Journal publication and/or registry report	86 (85)
	**Date of CSR**	1989	2 (2)
		1990–1994	12 (12)
		1995–1999	20 (20)
		2000–2004	40 (40)
		2005–2009	26 (26)
		2010	1 (1)
**Outcome characteristics**	**Total number of outcomes in sample** e		1,080 (100)
	**Benefit outcomes**		456 (42)
		Mortality	92 (9)
		Clinical events	119 (11)
		Symptoms	215 (20)
		HRQoL	30 (3)
	**Harm outcomes**		624 (58)
		AE	101 (9)
		SAE	101 (9)
		Withdrawal due to AE	101 (9)
		AE of special interest	321 (30)

a Premarketing: Phases II-IIIa; post-marketing: Phase IIIb and IV.

b Trial with primary efficacy outcome.

c Trial with primary safety outcome.

d Website no longer available.

e The 1,080 outcomes represent all patient-relevant outcomes reported in CSRs, journal publications, or registry reports on the 101 eligible trials (i.e., trials with a full CSR) included in 16 HTAs. All outcomes are mutually exclusive.

**Table 2:** Coding of completeness of reporting of trial outcomes.

Category of Completeness	Completely Reported including Numerical Data	Partly Reported including Numerical Data	Verbally Reported without Numerical Data	Not Reported
**General definitions**	Continuous data:• For end-of-trial value or for change from baseline: estimated effect size and confidence interval or standard errorCategorical data:• Number of patients with events and percent of analysis dataset per group• Percent not required if total number of patients in analysis dataset clearly available	• Any of the items of full reporting missing• Data insufficient to be included in meta-analysis (without derivation of data, e.g., of variance from *p*-values)	• Verbal reporting of statistical significance of group difference (statistically significant/not statistically significant)	• No information on outcome (trial document not available or outcome not published)• Verbal description without information on statistical significance of group difference (e.g., “no differences,” “comparable results”)• Results only available graphically in a figure without reporting of exact numerical data• Number of patients with an event (e.g., patients with minor hypoglycemia) without definition of the event
**Additional specific definitions**				
Patients with AEs		• Reporting of the percent of patients with AEs without the number of patients affected by AEs		• Reporting of numbers of AEs without the number of patients affected by AE• no data on AEs
Patients with AEs of special interest		• Reporting of the percent of patients with AEs of special interest without the number of patients affected by AEs of special interest		• Reporting of numbers of AEs of special interest without the number of patients affected by AEs of special interest• no data on AEs of special interest
Patients with SAEs		• Reporting of the percent of patients with SAEs without the number of patients affected by SAEs		• Reporting of SAEs without the number of patients affected by SAEs• No data on SAEs
Patients withdrawn due to AEs		• Reporting of the percent of patients withdrawn due to AEs without the number of patients withdrawn due to AEs		• Reporting of AEs resulting in withdrawal with no data on patients withdrawn due to AEs

**Box 1. Coding of Outcome Categories**

**Mortality (benefit outcome):** Any event/complication of the disease resulting in death, i.e., overall mortality and event-specific mortality, e.g., fatal myocardial infarction in diseases in which myocardial infarction is a late complication of the disease.

**Clinical events (benefit outcome):** Any event (other than an AE) based on a clinical diagnosis, e.g., nonfatal stroke or nonfatal myocardial infarction (if complication of investigated disease), asthma exacerbation.

**Symptoms (benefit outcome):** Any signs of the disease based on the description by the patient, e.g., asthma symptoms, pain, symptoms of depression.

**Health-related quality of life (benefit outcome):** Trial outcomes based on multidimensional questionnaires describing the impact of the disease and its treatment on physical, psychological, and social functioning and well-being, e.g., outcomes based on the Short Form 36 Questionnaire or the Asthma Quality of Life Questionnaire.

**AE categories (harm outcome):** Trial outcomes specified as AEs, SAEs, or withdrawals due to AEs based on the definitions used in the CSRs (usually according to definitions for clinical safety data management according to the International Conference on Harmonisation).

Our requirements for complete reporting of patient-relevant outcomes were based on the requirements of authors of systematic reviews (i.e., provision of adequate information for assessment of risk of bias and adequate data for meta-analyses) [24]. Completeness of the information provided for the patient-relevant outcomes was recorded as (1) completely reported including numerical data, (2) partly reported including numerical data, (3) verbally reported without numerical data, or (4) not reported. A definition of all categories is provided in Table 2. In the assessment of completeness of information in journal publications, if more than one journal publication was available and an outcome was completely reported in one publication but not in the other(s), reporting of the outcome was still classified as “complete.”

All data for the current study were extracted and coded by one author. All data from registry reports and all classifications of patient-relevant outcomes were independently checked by a second author. In addition, a random sample of 10% of the data and codings for trial outcomes from CSRs and journal publications was also independently checked by a second author (agreement between authors for CSRs: 99%; for journal publications: 97%). Discrepancies were resolved by consensus, if necessary, after discussion with a third author.

To quantify the information gain through CSRs, we calculated the proportion of outcomes with complete reporting (category 1 above) and incomplete reporting (categories 2–4 above) for CSRs and publicly available sources (journal publications, registry reports, and the combination of both). Besides presenting the dichotomous categories “complete reporting” versus “incomplete reporting,” we also presented separately the three categories of incomplete reporting (categories 2–4 above). In addition, we performed direct comparisons of trials for which CSRs as well as journal publications and/or registry reports were available. To investigate completeness of reporting over time, we calculated the proportion of outcomes with complete reporting in the different document types stratified by year of finalization of the CSRs.

The proportion of outcomes with complete reporting was compared between CSRs and journal publications or registry reports (or a combination of both) using the McNemar test to take the potential dependency of samples into account. The data were analyzed using SAS 9.2.

The manuscripts of the previous and current study show a minor overlap of results data: as AEs were investigated under the research questions of both studies, both manuscripts report the proportion of outcomes with complete information in CSRs for AEs (92%), SAEs (88%), and withdrawals due to AEs (91%) (see Table 3).

**Table 3:** Completeness of information for trial outcomes in CSRs, registry reports, and journal publications.

Type of Outcome	Number of Outcomes	Outcomes with Complete Information, *n* (Percent^a^)			
		Not Publicly Available	Publicly Available		
		CSR^b^ (*n* = 101)	Journal Publication and/or Registry Report^c^ (*n* = 86)	Journal Publication Only (*n* = 65)	Registry Report^c^ Only (*n* = 50)
**All outcomes** d	1,080	930 (86)	425 (39)	250 (23)	242 (22)
**Benefit outcomes**	456	385 (84)	158 (35)	88 (19)	88 (19)
Mortality	92	92 (100)	49 (53)	28 (30)	30 (33)
Clinical events	119	108 (91)	38 (32)	32 (27)	8 (7)
Symptoms	215	168 (78)	65 (30)	26 (12)	46 (21)
HRQoL	30	17 (57)	6 (20)	2 (7)	4 (13)
**Harm outcomes**	624	545 (87)	267 (43)	162 (26)	154 (25)
AEs	101	93 (92)	55 (54)	21 (21)	41 (41)
SAEs	101	89 (88)	52 (51)	24 (24)	37 (37)
Withdrawal due to AEs	101	92 (91)	73 (72)	51 (51)	42 (42)
Special AEs^e^	321	271 (84)	87 (27)	66 (21)	34 (11)

Trial sample: all studies with a CSR.

a Total number of outcomes with complete information/total number of corresponding outcomes in sample.

b CSRs submitted to regulatory authorities.

c Reports posted in trial results registries.

d All outcomes are mutually exclusive.

e AEs of special interest in the given indication.

## Results

Table 1 shows the characteristics of the trials, documents, and patient-relevant outcomes included in our sample. We analyzed 101 trials with CSRs. These CSRs were prepared between 24 September 1989 and 29 January 2010. The pool of clinical trials included nearly 70,000 patients and covered six different therapeutic areas (mainly depression and type I and II diabetes; the drugs assessed are listed by therapeutic area in Table 4). Of the 101 trials, 90 were efficacy trials; 86 had at least one publicly available source, 65 had at least one journal publication, and 50 had a registry report. For 15 trials, the CSR was the only source of information available.

**Table 4:** Therapeutic areas and drugs investigated in the CSRs, as well as missing outcomes in publicly available sources.

Therapeutic Area (Number of Trials)	Drugs Assessed	Examples of Patient-Relevant Trial Outcomes Not Reported in Publication or Registry Report (but Available in CSR)
Depression (*n* = 40)	Bupropion, duloxetine, mirtazapine, reboxetine, venlafaxine	Mortality: overall mortalitySymptoms: depression (MADRS, HAMD), cognition (MMSE), pain (VAS), anxiety (HAMA)HRQoL: QLDS, Q-LES-Q, SF36AEs: overall rate of AEs, SAEs, withdrawal due to AEs, special AEs (suicidal behavior, sexual dysfunction [ASEX, CSFQ])
Type II diabetes (*n* = 30)	Insulin detemir, insulin glargine, insulin glulisine, insulin lispro, nateglinide, pioglitazone, repaglinide, rosiglitazone	Mortality: overall mortality, cardiovascular mortalityClinical events: retinopathy, nonfatal myocardial infarction, stroke, severe hyperglycemiaHRQoL: W-BQ, DHP-18AEs: overall rate of AEs, overall rate of SAEs, withdrawal due to AEs, special AEs (cardiac SAEs, cerebral SAEs, severe hypoglycemia [at night], edema, injection site reaction)
Type I diabetes (*n* = 14)	Insulin aspart, insulin glulisine, insulin lispro	Mortality: overall mortality, combined outcomes including mortality components (e.g., fatal myocardial infarction)Clinical events: retinopathy, severe hyperglycemic eventHRQoL: W-BQ, DQOLY, DTSQAEs: overall rate of AEs, overall rate of SAEs, withdrawal due to AEs, special AEs (severe hypoglycemia [at night], injection site reaction)
Asthma (*n* = 9)	Beclometasone/formoterol, formoterol/budesonide, montelukast, salmeterol/fluticasone	Clinical events: asthma exacerbationSymptoms: asthma symptoms, sleep scores, symptom-free days and nightsAEs: overall rate of AEs
Stroke/transient ischemic attack (*n* = 5)	Dipyridamole+acetylsalicylic acid	Mortality: overall mortality, fatal stroke, vascular deathClinical events: nonfatal stroke, transient ischemic attackSymptoms: cognition (MMSE)HRQoL: EQ-5DAEs: overall rate of AEs, overall rate of SAEs, withdrawal due to AEs, special AEs (major and minor bleeding)
Alzheimer disease (*n* = 3)	Memantine	Symptoms: concomitant psychopathological symptoms, cognitive function, daily activities

ASEX, Arizona Sexual Experience Scale; CSFQ, Changes in Sexual Functioning Questionnaire; DHP-18, Diabetes Health Profile; DQOLY, Diabetes Quality of Life Questionnaire for Youth; DTSQ, Diabetes Treatment Satisfaction Questionnaire; EQ-5D, EuroQol-5D; HAMA, Hamilton Anxiety Scale; HAMD, Hamilton Depression Rating Scale; MADRS, Montgomery–Asberg Depression Rating Scale; MMSE, Mini Mental State Examination; QLDS, Quality of Life in Depression Scale; Q-LES-Q, Quality of Life Enjoyment and Satisfaction Questionnaire; SF36, Short Form 36; VAS, Visual Analogue Scale; W-BQ, Well-Being Questionnaire.

The 101 trials included 1,080 outcomes classified by IQWiG as patient-relevant and considered in the pool of HTAs. Among the benefit outcomes, symptoms were investigated most often, whereas HRQoL was investigated least often. Among the harm outcomes, overall rates of AEs, SAEs, and withdrawals due to AEs were available for each trial; the harm outcomes considered most often were AEs of special interest in the given indication.

### Overall Completeness of Information in Clinical Study Reports versus Publicly Available Sources

Table 3 shows the completeness of information for trial outcomes by reporting format in the full trial sample. The CSRs provided complete information on a considerably higher proportion of patient-relevant outcomes (86%) than journal publications and registry reports, even if these two sources were combined (39%). With the exception of HRQoL (57%), CSRs provided complete information on 78% to 100% of benefit outcomes. The highest value was achieved for mortality (100%). The corresponding values for combined publicly available sources were considerably lower; completeness of reporting ranged from 20% to 53%. CSRs provided complete information on 84% to 92% of harm outcomes. Again, the corresponding values for the combined publicly available sources were considerably lower (27% to 72%). The comparison of journal publications and registry reports showed that, overall, completeness of information was similar for benefit outcomes (19%) and harm outcomes (25% to 26%). However, when specific outcomes were considered, the two reporting formats showed different levels of completeness (e.g., for clinical events or the overall rate of AEs).

The differences in completeness of information for patient-relevant outcomes between CSRs and journal publications or registry reports (or a combination of both) were statistically significant for all types of outcomes (see Table 5).

**Table 5:** Comparison of proportions of outcomes with complete information (matched pairs; McNemar test) (sample: all trials with a CSR; *n* = 101).

Type of Outcome	Number of Outcomes	Discordant Pairs and *p*-Values for CSRs^a^ versus Publicly Available Sources		
		Journal Publication and/or Registry Report^b^: *n*_csr_ (Percent)/*n*_jp,reg_ (Percent)	Journal Publication Only: *n*_csr_ (Percent)/*n*_jp_ (Percent)	Registry Report^b^ Only: *n*_csr_ (Percent)/*n*_reg_ (Percent)
**All outcomes**	1,080	515 (48)/10 (1)<0.001	688 (64)/8 (1)<0.001	691 (64)/3 (<1)<0.001
**Benefit outcomes**	456	231 (51)/4 (1)<0.001	300 (66)/3 (1)<0.001	298 (65)/1 (<1)<0.001
Mortality	92	43 (47)/0<0.001	64 (70)/0<0.001	62 (67)/0<0.001
Clinical events	119	74 (62)/4 (3)<0.001	79 (66)/3 (3)<0.001	101 (85)/1 (1)<0.001
Symptoms	215	103 (48)/0<0.001	142 (66)/0<0.001	122 (57)/0<0.001
HRQoL	30	11 (37)/0<0.001	15 (50)/0<0.001	13 (43)/0<0.001
**Harm outcomes**	624	284 (46)/6 (1)<0.001	388 (62)/5 (1)<0.001	393 (63)/2 (<1)<0.001
AEs	101	38 (38)/0<0.001	72 (71)/0<0.001	52 (51)/0<0.001
SAEs	101	38 (38)/1 (1)<0.001	65 (64)/0<0.001	53 (52)/1 (1)<0.001
Withdrawal due to AEs	101	21 (21)/2(2)<0.001	43 (43)/2 (2)<0.001	50 (50)/0<0.001
Special AEs^c^	321	187 (58)/3 (1)<0.001	208 (65)/3 (1)<0.001	238 (74)/1 (<1)<0.001

a CSRs submitted to regulatory authorities.

b Reports posted in trial results registries.

c AEs of special interest in the given indication.

*n*_csr_, number of outcomes where complete information is provided by the CSR but not by the journal publication and/or registry report; *n*_jp,reg_, number of outcomes where complete information is provided by the journal publication and/or registry report but not by the CSR; *n*_jp_, number of outcomes where complete information is provided by the journal publication but not by the CSR; *n*_reg_, number of outcomes where complete information is provided by the registry report but not the CSR.

### Publication Bias and Outcome Reporting Bias

In addition to analyzing the proportion of outcomes for which complete information was available, we also aimed to further describe the reporting of outcomes with incomplete information. Table 6 presents the pattern of reporting of all patient-relevant outcomes in journal publications and/or registry reports. The data show that most outcomes that were not reported completely were not reported at all, except for outcomes on symptoms and HRQoL, which were reported partly with data or only verbally without data in 35% to 40% of cases. However, also for these two outcomes a large proportion of outcomes were not available at all from publicly available sources. Non-availability of outcomes was due either to lack of reporting of these outcomes even though a publication and/or registry report was available (34% of all outcomes, outcome reporting bias) or to lack of reporting of the whole trial (13%, publication bias). Tables S1 and S2 show the same type of analysis for journal publications and registry reports separately. Table 4 provides examples of the numerous patient-relevant outcomes (including outcomes of major clinical relevance, such as overall mortality and potentially life-threatening events) not reported in the publicly available sources, by therapeutic area and outcome category.

**Table 6:** Pattern of reporting of trial outcomes in journal publications and/or registry reports.

Type of Outcome	Number of Outcomes	Extent of Reporting of Outcomes in Journal Publications and/or Registry Reports^a^, *n* (Percent^b^)				
		Reported Completely	Reported Partly with Data	Reported Verbally without Data	Not Reported in Publication/Registry Report of Trial	Neither Publication nor Registry Report Available for Trial
**All outcomes**	1,080	425 (39)	133 (12)	17 (2)	368 (34)	137 (13)
**Benefit outcomes**	456	158 (35)	84 (18)	13 (3)	142 (31)	59 (13)
Mortality	92	49 (53)	2 (2)	0	26 (28)	15 (16)
Clinical events	119	38 (32)	5 (4)	4 (3)	67 (56)	5 (4)
Symptoms	215	65 (30)	70 (33)	4 (2)	38 (18)	38 (18)
HRQoL	30	6 (20)	7 (23)	5 (17)	11 (37)	1 (3)
**Harm outcomes**	624	267 (43)	49 (8)	4 (1)	226 (36)	78 (13)
AEs	101	55 (54)	7 (7)	1 (1)	23 (23)	15 (15)
SAEs	101	52 (51)	4 (4)	0	30 (30)	15 (15)
Withdrawal due to AEs	101	73 (72)	4 (4)	0	9 (9)	15 (15)
Special AEs^c^	321	87 (27)	34 (11)	3 (1)	164 (51)	33 (10)

Trial sample: all studies with a CSR.

a Reports posted in trial results registries.

b Total number of outcomes with complete information/total number of corresponding outcomes in sample.

c AEs of special interest in the given indication.

### Matched Pairs of Clinical Study Reports versus Publications and/or Registry Reports

The results presented so far describe the completeness of information in publicly available reporting formats for a given sample of trials (101 trials with CSRs). Part of the differences described resulted from the fact that journal publications or registry reports were not available for all trials in our sample, as it also included unpublished trials only reported in CSRs. To investigate whether CSRs provided superior information when they were directly compared to the corresponding journal publications and registry reports, we analyzed the completeness of information in CSRs versus the publicly available sources in samples including only trials for which the respective source was available (see Tables 7 and S3–S5). Overall, each of these analyses confirmed that a substantial amount of additional information on patient-relevant outcomes is gained from CSRs compared to journal publications or registry reports (or a combination of both), even for published trials.

**Table 7:** Analysis of completeness of information for trial outcomes in CSRs versus publicly available sources, i.e., registry reports and/or journal publications.

Type of Outcome	Number of Outcomes	Outcomes with Complete Information, *n* (Percent^a^)	
		Not Publicly Available: CSR^b^ (*n* = 86)	Publicly Available: Registry Report^c^ and/or Journal Publication (*n* = 86)
**All outcomes**	943	822 (87)	425 (45)
**Benefit outcomes**	397	340 (86)	158 (40)
Mortality	77	77 (100)	49 (64)
Clinical events	114	103 (90)	38 (33)
Symptoms	177	144 (81)	65 (37)
HRQoL	29	16 (55)	6 (21)
**Harms outcomes**	546	482 (88)	267 (49)
AEs	86	82 (95)	55 (64)
SAEs	86	76 (88)	52 (60)
Withdrawal due to AEs	86	81 (94)	73 (85)
Special AEs^d^	288	243 (84)	87 (30)

Sample: all trials with both a CSR and a registry report and/or journal publication, *n* = 86).

a Total number of outcomes with complete information/total number of respective outcomes in sample.

b CSRs submitted to regulatory authorities.

c Reports posted in trial results registries.

d AEs of special interest in the given indication.

### Completeness of Reporting over Time

To investigate completeness of reporting over time, we analyzed the availability of trial reports in publicly available sources as well as the proportion of completely reported outcomes in the three document types over time (Table 8). The analysis showed an increasing availability of trials in combined publicly available sources over time (from 71% to 95%). However, the proportion of trials available in journal publications dropped to about 50% for trials with CSRs finalized between 2005 and 2010. We hypothesized that this decrease could have been caused by the fact that the temporal proximity of the literature searches in the HTAs and the finalization date of the CSR had not allowed sufficient time for preparation and publication of a manuscript. We therefore performed a sensitivity analysis in which all trials with a CSR finalization date less than 2 y before the search date of the HTA were classified as published in a journal. This “best-case scenario” resulted in an availability rate of trials in journal publications of 81%. We also performed the same type of analysis for registry reports; the corresponding rate was 93%.

**Table 8:** Availability of trials and completeness of information for patient-relevant outcomes in publicly available sources by year of CSR.

Year of Finalization of the CSR	Not Publicly Available		Publicly Available					
	CSR^a^ (*n* = 101)		Journal Publication and/or Registry Report^b^ (*n* = 86)		Journal Publication Only (*n* = 65)		Registry Report^b^ Only (*n* = 50)	
	Outcomes per sample^c^, *n* (Number of Trials, Percent)^d^	Outcomes with Complete Information, *n* (Percent^e^)	Outcomes per Sample^c^, *n* (Number of Trials, Percent)^d^	Outcomes with Complete Information, *n* (Percent^e^)	Outcomes per Sample^c^, *n* (Number of Trials, Percent)^d^	Outcomes with Complete Information, *n* (Percent^e^)	Outcomes per Sample^c^, *n* (Number of Trials, Percent)^d^	Outcomes with Complete Information, *n* (Percent^e^)
All (1989–2010)	1,080 (101 trials, 100%)	930 (86)	943 (86 trials, 85%)	425 (45)	724 (65 trials, 64%)	250 (35)	535 (50 trials, 50%)	242 (45)
1989–1994	109 (14 trials, 100%)	43 (39)	77 (10 trials, 71%)	20 (26)	41 (5 trials, 36%)	3 (7)	45 (6 trials, 43%)	17 (38)
1995–1999	209 (20 trials, 100%)	193 (92)	184 (17 trials, 85%)	82 (45)	184 (17 trials, 85%)	68 (37)	43 (5 trials, 25%)	21 (49)
2000–2004	424 (40 trials, 100%)	385 (91)	367 (34 trials, 85%)	176 (48)	317 (29 trials, 73%)	92 (29)	218 (20 trials, 50%)	117 (54)
2005–2010	338 (27 trials, 100%)	309 (91)	315 (25 trials, 93%)	147 (47)	182 (14 trials, 52%^f^)	87 (48)	229 (19 trials, 70%^g^)	87 (38)

a CSRs submitted to regulatory authorities.

b Reports posted in trial results registries.

c Number of all patient-relevant outcomes available in the trials included in the given document pool (per time period).

d Number of all trials included in the given document pool (per time period); percent of all trials from a given time period.

e Total number of outcomes with complete information/total number of outcomes available in the trials included in the given document pool (per time period).

f For eight trials the time from finalization of the CSR to bibliographic search was <2 y (three with <1 y and five with >1 and <2 y, all in 2005–2010); a sensitivity analysis in which these trials were classified as having a publication resulted in a rate of 81% of trials with journal publications.

g For six trials the time from finalization of the CSR to the search in registries was <2 y (three with <1 y, three with >1 and <2 y, all in 2005–2010); a sensitivity analysis in which these trials were classified as having a registry report resulted in 93% of trials with registry reports.

However, in our sample, high availability rates of trial reports in publicly available sources did not result in high rates of completely reported patient-relevant outcomes: for instance, even for trials for which the availability rate in combined publicly available sources was more than 90%, less than 50% of patient-relevant outcomes were completely reported. In contrast, after 1995, CSRs consistently provided complete information for more than 90% of patient-relevant outcomes.

## Discussion

### Summary of Findings

To our knowledge the current study quantifies for the first time how much information on a wide range of patient-relevant outcomes included in a large pool of clinical trials can be gained from making full CSRs available. Our findings show that a substantial amount of information on patient-relevant outcomes required for unbiased trial evaluation is missing from the public record. This is all the more important as such outcomes are preferably considered in comparative effectiveness research and consequently in health policy and clinical decision-making [25,26]. At the same time, this information can be obtained from CSRs, i.e., from documents routinely prepared by sponsors of clinical trials, but not usually made publicly available. Over twice as much information on patient-relevant outcomes can be gained from CSRs than from publicly available sources (86% versus 39% completely reported outcomes). Moreover, CSRs not only provide patient-relevant information in cases where journal publications and registry reports are missing, they also present additional information in cases where trials have been reported in journals or registries. The differences in information gain from the different reporting formats are due to a general superiority of CSRs over publicly available sources, demonstrated by the higher proportion of completely reported outcomes in a matched sample of CSRs and publicly available documents. Our findings also again confirm the existence of considerable publication and outcome reporting bias in clinical research: 36% of the trials in our pool were not published in journal publications, 15% had no publicly available reports at all, and even for trials with publicly available reports, 34% of patient-relevant outcomes, including outcomes of major clinical relevance, were not reported.

Our analysis of completeness of reporting over time showed that although the rate of trials made available in journal publications and registry reports is increasing, the rate of completeness of information on patient-relevant outcomes in these sources is not. These findings show that new approaches are needed. It is insufficient to aim for a journal publication rate of 100%. What is needed is public availability of CSRs, and thus of documents presenting trial results to a level of detail required for full evaluation of a trial.

### Comparison with Previous Research

Because of the fact that CSRs are generally not publicly available, only a few researchers have investigated their content as well as their possible role in providing information on clinical trials. Doshi and Jefferson analyzed a sample of 78 CSRs and showed that CSRs had a median length of about 450 pages of text and main tables plus an additional 550 pages of efficacy and safety listings [27]. Vedula et al. compared unpublished internal company documents from the gabapentin litigation case (unpublished protocols, statistical analysis plans, and research reports) with trial publications [28,29]. Besides identifying several inconsistencies in the corresponding trial publications, they found that the unpublished documents provided more extensive documentation of methods planned and used, as well as trial findings. The research already cited analyzing CSRs on oseltamivir (Tamiflu) and reboxetine showed that prior conclusions on a drug's benefits and harms based on published evidence alone could no longer be upheld when information from CSRs became available [12,13]. Our previous study of CSRs showed that considerably more relevant information on trial methods, primary outcomes, and some AE outcomes can be gained from CSRs [23]. In the current study, information gain from CSRs versus publicly available sources was even higher for a full set of patient-relevant outcomes than for the limited set of trial outcomes investigated in our previous study. While the proportion of completely reported primary and AE outcomes in the previous study was 91% for CSRs, 52% for journal publications, and 71% for registry reports [23], the corresponding values for the full range of (primary and non-primary) patient-relevant outcomes investigated in the current study were 86%, 23%, and 22%, respectively.

### Relevance of Full Trial Information for Everyday Patient Care

Our findings suggest that oseltamivir and reboxetine might not be the only cases in which conclusions on benefits and harms might be changed by making full information on all clinical trials available to independent researchers and subsequently to clinicians and patients. Access to CSRs would thus allow informed decision-making and directly influence patient care.

The goal of assessing the full information from CSRs is not only to determine the benefits and harms of a single drug, but also to investigate the position of a drug in the given therapeutic area. For this purpose, comparative effectiveness research is gaining momentum both in the US and in Europe [25,30]. This area of research would specifically benefit from full CSRs being publicly available. As direct comparisons of alternative treatment methods are not available for all comparative effectiveness research questions, indirect comparisons will become more important, and CSRs are essential sources to inform meaningful indirect comparisons. This is because, firstly, indirect comparisons require detailed information on methods (i.e., a full protocol) of the clinical trials of interest, as well as on the trial population, to assess whether indirect comparisons within a given pool of trials are appropriate at all; this type of information is available in CSRs. Secondly, indirect comparisons require full numerical information on all relevant outcomes for network meta-analyses; as our analyses show, such extensive information is provided only in CSRs.

### How Can Full Access to Clinical Study Reports Be Achieved?

As stated, the EMA intends to proactively publish complete clinical trial data, possibly including CSRs, from January 2014 onwards [18], and for this purpose has held extensive consultations with advisory groups of stakeholders and other interested parties [19] and has published a draft policy [20]. An even clearer solution would be a legal requirement to make CSRs publicly available, as is currently being discussed for the planned European legislation on clinical research [22].

However, both initiatives have a potential major flaw: they probably would apply only to drugs approved from January 2014 onwards, or for trials conducted after new legislation came into effect. This would present a problem because most drugs in current use would not be covered by the new measures, yet these drugs will still be widely used in clinical practice for years to come. Thus, although comprehensive information would in future be available for newer drugs, published information on the majority of drugs would still remain biased. This would hamper a meaningful comparison of alternative treatment methods. In addition, open questions about drugs in current use may never be answered. This is particularly relevant for drugs with a substantial public health impact, such as oseltamivir. The CSRs in our pool of trials were prepared between 1985 and 2010 and prove the value of CSRs for drugs in current use. The CSRs of such drugs that were submitted to regulatory authorities should therefore be made publicly available in a central repository to complete the evidence base. Pharmaceutical companies and non-industry trial sponsors could also release CSRs, thus underlining their commitment to transparency.

In line with our point of view, a further initiative to promote trial registration and reporting of full methods and results, the AllTrials initiative (http://www.alltrials.net/), also specifically refers to “past and present” clinical trials.

### Further Rocks on the Road to Full Data Transparency

It should be noted that the full implementation of the new EMA policy is in jeopardy as the pharmaceutical industry, which has previously expressed its reservations about the policy [31], is taking legal action: two Freedom of Information requests were made to the EMA under its current data transparency policy to release individual patient data for adalimumab (Humira), a tumor necrosis factor inhibitor approved for rheumatoid arthritis and other indications. However, the company AbbVie has sought an injunction to block the EMA from releasing the data. A second company, Intermune, has also taken legal action against the EMA [32]. The interim decision by the General Court of the European Union is in favor of the companies: the EMA has been ordered not to provide documents until a final ruling is given by the Court [33]. The two court cases seem to represent not just the policy of single companies but a general industry strategy, since both European and US pharmaceutical industry bodies have lodged supportive pleas [32]. This action contradicts repeated assertions by industry that it supports data transparency.

### Limitations

Our study has a number of limitations. First of all, we were not able to investigate a representative or random sample of CSRs, because these documents are usually not available outside pharmaceutical companies and regulatory agencies. Therefore, our sample was based on CSRs provided voluntarily by pharmaceutical companies upon request during our assessment procedures. We did not receive a (full) CSR for 62% (167/268) of the trials included in our HTAs and thus had to exclude these trials from our current study. The excluded and included trials showed differences in the therapeutic areas investigated (see Table S6), for example, the former comprised a higher proportion of trials on depression (57% versus 40%), but a lower proportion of trials on diabetes (21% versus 44%). In addition, a higher proportion of the excluded trials were reported in journal publications (76% versus 65%), whereas a lower proportion of these trials were reported in registry reports (17% versus 50%). It is unclear whether our results would have been different if they had been based on a random sample of CSRs. Furthermore, our sample covered only a limited number of therapeutic areas and was restricted to randomized controlled trials investigating drugs, so we cannot comment on other trial designs or trials of non-drug interventions. In addition, the registry reports included were generated by a limited number of pharmaceutical companies and were not prepared according to the requirements of the Food and Drug Administration Amendments Act [23]; future reports in ClinicalTrials.gov may be of better quality.

The dataset for our study was generated in 2011. We did not perform an update of the dataset as this would have required a major investment of resources. However, we believe that this dataset, which includes a total of 101 trials with 1,080 patient-relevant outcomes, is large enough to produce meaningful results.

We also note that several further issues related to CSRs could be investigated in future research. For example, except for the case of reboxetine [13], we can make statements only about the completeness of information in CSRs versus publicly available sources; we did not analyze how often the inclusion of data from CSRs changed the interpretation of the overall results of a study. Moreover, we did not investigate whether certain study characteristics (e.g., enrollment size) influenced completeness of reporting, nor did we specifically describe study protocols included in CSRs.

### Conclusion

Information on patient-relevant outcomes investigated in clinical trials is insufficient in publicly available sources; considerably more information can be gained from CSRs. CSRs should be made publicly available as they may substantially influence conclusions concerning the actual position of an individual drug in a therapeutic area. Our findings underline the importance of CSRs—both for past and future trials—for unbiased trial evaluation, thus supporting informed decision-making in health care.

## Supporting Information

Table S1 **Pattern of reporting of trial outcomes in journal publications (sample: all trials with a CSR,** ***n*** **= 101).**
(DOC)

Table S2 **Pattern of reporting of trial outcomes in registry reports (sample: all trials with a CSR;** ***n*** **= 101).**
(DOC)

Table S3 **Analysis of completeness of information for trial outcomes in CSRs versus journal publications (sample: all trials with both a CSR and a journal publication;** ***n*** **= 65).**
(DOC)

Table S4 **Analysis of completeness of information for trial outcomes in CSRs versus registry reports (sample: all trials with both a CSR and a registry report;** ***n*** **= 50).**
(DOC)

Table S5 **Analysis of completeness of information for trial outcomes in CSRs versus the combination of journal publications and registry reports (sample: all trials with a CSR and a registry report and a journal publication;** ***n*** **= 29).**
(DOC)

Table S6 **Characteristics of excluded trials and documents.**
(DOC)

## Abbreviations

AE: adverse event
CSR: clinical study report
EMA: European Medicines Agency
HRQoL: health-related quality of life
HTA: health technology assessment
IQWiG: Institute for Quality and Efficiency in Health Care
SAE: serious adverse event
