# Supplementary material for: Completeness of Reporting of Patient-Relevant Clinical Trial Outcomes: Comparison of Unpublished Clinical Study Reports with Publicly Available Data
Source: PLoS Med. 2013 Oct 8;10(10):e1001526. doi: 10.1371/journal.pmed.1001526 (PMC3793003; doi:10.1371/journal.pmed.1001526)
Supplement: Table S1 — Pattern of reporting of trial outcomes in journal publications (sample: all trials with a CSR, n = 101). (DOC) [file pmed.1001526.s001.doc]

Table S1: Pattern of reporting of trial outcomes in journal publications (sample: all trials with a CSR, N=101)

| Type of outcome | No. of out-comes | Extent of reporting of outcomes in journal publications, n (%^a^) | | | | |
| --- | --- | --- | --- | --- | --- | --- |
|  |  | Reported completely | Reported partly with data | Reported verbally without data | Not reported | No publication available for trial |
| **All outcomes** | **1080** | **250 (23)** | **128 (12)** | **11 (1)** | **335 (31)** | **356 (33)** |
| **Benefit outcomes** | **456** | **88 (19)** | **86 (19)** | **10 (2)** | **136 (30)** | **136 (30)** |
| Mortality | 92 | 28 (30) | 2 (2) | 0 | 30 (33) | 32 (35) |
| Clinical event | 119 | 32 (27) | 5 (4) | 2 (2) | 49 (41) | 31 (26) |
| Symptom | 215 | 26 (12) | 74 (34) | 6 (3) | 44 (20) | 65 (30) |
| HRQoL | 30 | 2 (7) | 5 (17) | 2 (7) | 13 (43) | 8 (27) |
| Harm outcomes | **624** | **162 (26)** | **42 (7)** | **1 (<1)** | **199 (32)** | **220 (35)** |
| AE | 101 | 21 (21) | 9 (9) | 0 | 35 (35) | 36 (36) |
| SAE | 101 | 24 (24) | 3 (3) | 1 (1) | 37 (37) | 36 (36) |
| Withdrawal due to AE | 101 | 51 (51) | 5 (5) | 0 | 9 (9) | 36 (36) |
| Special AE^b^ | 321 | 66 (21) | 25 (8) | 0 | 118 (37) | 112 (35) |

a: Total number of outcomes with complete information/ total number of corresponding outcomes in sample

b: Adverse events of special interest in the given indication

AE: adverse event; CSR: clinical study report; HRQoL: health-related quality of life; n: number of outcomes with specified information; SAE: serious adverse event
