# Supplementary material for: Completeness of Reporting of Patient-Relevant Clinical Trial Outcomes: Comparison of Unpublished Clinical Study Reports with Publicly Available Data
Source: PLoS Med. 2013 Oct 8;10(10):e1001526. doi: 10.1371/journal.pmed.1001526 (PMC3793003; doi:10.1371/journal.pmed.1001526)
Supplement: Table S2 — Pattern of reporting of trial outcomes in registry reports (sample: all trials with a CSR; n = 101). (DOC) [file pmed.1001526.s002.doc]

Table S2: Pattern of reporting of trial outcomes in registry reports (sample: all trials with a CSR; N=101)

| Type of outcome | No. of out-comes | Extent of reporting of outcomes in registry reports^a^, n (%^b^) | | | | |
| --- | --- | --- | --- | --- | --- | --- |
|  |  | Reported completely | Reported partly with data | Reported verbally without data | Not reported | No registry report available for trial |
| **All outcomes** | **1080** | **242 (22)** | **69 (6)** | **14 (1)** | **210 (19)** | **545 (50)** |
| **Benefit outcomes** | **456** | **88 (19)** | **49 (11)** | **9 (2)** | **84 (18)** | **226 (50)** |
| Mortality | 92 | 30 (33) | 1 (1) | 0 | 8 (9) | 53 (58) |
| Clinical event | 119 | 8 (7) | 2 (2) | 2 (2) | 34 (29) | 73 (61) |
| Symptom | 215 | 46 (21) | 38 (18) | 2 (1) | 37 (17) | 92 (43) |
| HRQoL | 30 | 4 (13) | 8 (27) | 5 (17) | 5 (17) | 8 (27) |
| Harm outcomes | **624** | **154 (25)** | **20 (3)** | **5 (1)** | **126 (20)** | **319 (51)** |
| AE | 101 | 41 (41) | 1 (1) | 1 (1) | 7 (7) | 51 (51) |
| SAE | 101 | 37 (37) | 3 (3) | 0 | 10 (10) | 51 (51) |
| Withdrawal due to AE | 101 | 42 (42) | 3 (3) | 1 (1) | 4 (4) | 51 (51) |
| Special AE^c^ | 321 | 34 (11) | 13 (4) | 3 (1) | 105 (33) | 166 (52) |

a: Reports posted in trial results registries

b: Total number of outcomes with complete information/ total number of corresponding outcomes in sample

c: Adverse events of special interest in the given indication

AE: adverse event; CSR: clinical study report; HRQoL: health-related quality of life; n: number of outcomes with specified information;; SAE: serious adverse event
