# Supplementary material for: Completeness of Reporting of Patient-Relevant Clinical Trial Outcomes: Comparison of Unpublished Clinical Study Reports with Publicly Available Data
Source: PLoS Med. 2013 Oct 8;10(10):e1001526. doi: 10.1371/journal.pmed.1001526 (PMC3793003; doi:10.1371/journal.pmed.1001526)
Supplement: Table S3 — Analysis of completeness of information for trial outcomes in CSRs versus journal publications (sample: all trials with both a CSR and a journal publication; n = 65). (DOC) [file pmed.1001526.s003.doc]

Table S3: Analysis of completeness of information for trial outcomes in CSRs versus journal publications (sample: all trials with both a CSR and a journal publication; N=65)

| **Type of outcome** | **Number of outcomes** | **Outcomes with complete information, n (%^a^)** | |
| --- | --- | --- | --- |
|  |  | **Not publicly available** | **Publicly available** |
|  |  | **CSR^b^**  **(N = 65)** | **Journal publication**  **(N =65)** |
| **All outcomes** | **724** | **640 (88)** | **250 (35)** |
| **Benefit outcomes** | **320** | **275 (86)** | **88 (28)** |
| Mortality | 60 | 60 (100) | 28 (47) |
| Clinical event | 88 | 79 (90) | 32 (36) |
| Symptom | 150 | 122 (81) | 26 (17) |
| HRQoL | 22 | 14 (64) | 2 (9) |
| **Harms outcomes** | **404** | **365 (90)** | **162 (40)** |
| AE | 65 | 61 (94) | 21 (32) |
| SAE | 65 | 60 (92) | 24 (37) |
| Withdrawal due to AE | 65 | 61 (94) | 51 (78) |
| Special AE**^c^** | 209 | 183 (88) | 66 (32) |

a: Total number of outcomes with complete information / total number of respective outcomes in sample

b: CSRs submitted to regulatory authorities

c: Adverse events of special interest in the given indication

AE: adverse event; CSR: clinical study report; HRQoL: health-related quality of life; n: number of outcomes with complete information; SAE: serious adverse event
