# Supplementary material for: Completeness of Reporting of Patient-Relevant Clinical Trial Outcomes: Comparison of Unpublished Clinical Study Reports with Publicly Available Data
Source: PLoS Med. 2013 Oct 8;10(10):e1001526. doi: 10.1371/journal.pmed.1001526 (PMC3793003; doi:10.1371/journal.pmed.1001526)
Supplement: Table S4 — Analysis of completeness of information for trial outcomes in CSRs versus registry reports (sample: all trials with both a CSR and a registry report; n = 50). (DOC) [file pmed.1001526.s004.doc]

Table S4: Analysis of completeness of information for trial outcomes in CSRs versus registry reports (sample: all trials with both a CSR and a registry report; N=50)

| **Type of outcome** | **Number of outcomes** | **Outcomes with complete information, n (%^a^)** | |
| --- | --- | --- | --- |
|  |  | **Not publicly available** | **Publicly available** |
|  |  | **CSR^b^**  **(N = 50)** | **Registry reports^c^**  **(N = 50)** |
| **All outcomes** | **535** | **462 (86)** | **242 (45)** |
| **Benefit outcomes** | **230** | **191 (83)** | **88 (38)** |
| Mortality | 39 | 39 (100) | 30 (77) |
| Clinical event | 46 | 39 (85) | 8 (17) |
| Symptom | 123 | 103 (84) | 46 (37) |
| HRQoL | 22 | 10 (46) | 4 (18) |
| **Harm outcomes** | **305** | **271 (89)** | **154 (50)** |
| AE | 50 | 50 (100) | 41 (82) |
| SAE | 50 | 43 (86) | 37 (74) |
| Withdrawal due to AE | 50 | 49 (98) | 42 (84) |
| Special AE**^d^** | 155 | 129 (83) | 34 (22) |

a: Total number of outcomes with complete information / total number of respective outcomes in sample

b: CSRs submitted to regulatory authorities

c: Reports posted in trial results registries

d: Adverse events of special interest in the given indication

AE: adverse event; CSR: clinical study report; HRQoL: health-related quality of life; n: number of outcomes with complete information; SAE: serious adverse event
