# Supplementary material for: Completeness of Reporting of Patient-Relevant Clinical Trial Outcomes: Comparison of Unpublished Clinical Study Reports with Publicly Available Data
Source: PLoS Med. 2013 Oct 8;10(10):e1001526. doi: 10.1371/journal.pmed.1001526 (PMC3793003; doi:10.1371/journal.pmed.1001526)
Supplement: Table S5 — Analysis of completeness of information for trial outcomes in CSRs versus the combination of journal publications and registry reports (sample: all trials with a CSR and a registry report and a journal publication; n = 29). (DOC) [file pmed.1001526.s005.doc]

Table S5: Analysis of completeness of information for trial outcomes in CSRs versus the combination of journal publications and registry reports (sample: all trials with a CSR and a registry report and a journal publication; N=29)

| **Type of outcome** | **Number of outcomes** | **Outcomes with complete information, n (%^a^)** | | |
| --- | --- | --- | --- | --- |
|  |  | **Not publicly available** | | **Publicly available** |
|  |  | **CSR^b^**  **(N = 29)** | **Registry report^c^** **and journal publication**  **(N = 29)** | |
| **All outcomes** | **316** | **280 (89)** | **201 (64)** | |
| **Benefit outcomes** | **153** | **126 (82)** | **83 (54)** | |
| Mortality | 22 | 22 (100) | 19 (86) | |
| Clinical event | 20 | 15 (75) | 5 (25) | |
| Symptom | 96 | 81 (84) | 54 (56) | |
| HRQoL | 15 | 8 (53) | 5 (33) | |
| **Harms outcomes** | **163** | **154 (94)** | **118 (72)** | |
| AE | 29 | 29 (100) | 27 (93) | |
| SAE | 29 | 27 (93) | 25 (86) | |
| Withdrawal due to AE | 29 | 29 (100) | 27 (93) | |
| Special AE^d^ | 76 | 69 (91) | 39 (51) | |

a: Total number of outcomes with complete information/ total number of respective outcomes in sample

b: CSRs submitted to regulatory authorities during drug approval

c: Reports posted in trial results registries

d: Adverse events of special interest in the given indication

AE: adverse event; CSR: clinical study report; HRQoL: health-related quality of life; n: number of outcomes with complete information; SAE: serious adverse event
