# Supplementary material for: Completeness of Reporting of Patient-Relevant Clinical Trial Outcomes: Comparison of Unpublished Clinical Study Reports with Publicly Available Data
Source: PLoS Med. 2013 Oct 8;10(10):e1001526. doi: 10.1371/journal.pmed.1001526 (PMC3793003; doi:10.1371/journal.pmed.1001526)
Supplement: Table S6 — Characteristics of excluded trials and documents. (DOC) [file pmed.1001526.s006.doc]

Table S6: Characteristics of excluded trials and documents

| **Trial characteristics** | **Studies, N (%)** |
| --- | --- |
| **Trials excluded** | **167 (100)** |
| **Therapeutic area** |  |
| Depression | 96 (57) |
| Type II diabetes | 22 (13) |
| Type I diabetes | 14 (8) |
| Asthma | 26 (16) |
| Stroke/transient ischaemic attack | 1 (1) |
| Alzheimer’s disease | 7 (4) |
| Urology | 1 (1) |
| **Funding** |  |
| Industry funding | 132 (79) |
| Non-industry funding^a^ | 35 (21) |
| **Document type available** |  |
| Incomplete CSR | 89 (53) |
| Journal publication | 127 (76) |
| Registry report | 28 (17) |
| Journal publication and / or registry report | 137 (82) |

a: No funding by industry mentioned

CSR: clinical study report
